# Reconstruction of the Regulatory Network for *Bacillus subtilis* and Reconciliation with Gene Expression Data

**DOI:** 10.3389/fmicb.2016.00275

**Published:** 2016-03-18

**Authors:** José P. Faria, Ross Overbeek, Ronald C. Taylor, Neal Conrad, Veronika Vonstein, Anne Goelzer, Vincent Fromion, Miguel Rocha, Isabel Rocha, Christopher S. Henry

**Affiliations:** ^1^Computation Institute, University of ChicagoChicago, IL, USA; ^2^Computing, Environment and Life Sciences, Argonne National LaboratoryArgonne, IL, USA; ^3^Centre of Biological Engineering, University of MinhoBraga, Portugal; ^4^Fellowship for Interpretation of GenomesBurr Ridge, IL, USA; ^5^Computational Biology and Bioinformatics Group, Pacific Northwest National Laboratory, United States Department of EnergyRichland, WA, USA; ^6^UR1404 Applied Mathematics and Computer Science from Genomes to the Environment, INRA, Paris-Saclay UniversityJouy-en-Josas, France; ^7^Mathematics and Computer Science Division, Argonne National LaboratoryArgonne, IL, USA

**Keywords:** Atomic Regulon, regulatory network, stimuli, regulation, *Bacillus subtilis*

## Abstract

We introduce a manually constructed and curated regulatory network model that describes the current state of knowledge of transcriptional regulation of *Bacillus subtilis*. The model corresponds to an updated and enlarged version of the regulatory model of central metabolism originally proposed in 2008. We extended the original network to the whole genome by integration of information from DBTBS, a compendium of regulatory data that includes promoters, transcription factors (TFs), binding sites, motifs, and regulated operons. Additionally, we consolidated our network with all the information on regulation included in the SporeWeb and Subtiwiki community-curated resources on *B. subtilis*. Finally, we reconciled our network with data from RegPrecise, which recently released their own less comprehensive reconstruction of the regulatory network for *B. subtilis*. Our model describes 275 regulators and their target genes, representing 30 different mechanisms of regulation such as TFs, RNA switches, Riboswitches, and small regulatory RNAs. Overall, regulatory information is included in the model for ∼2500 of the ∼4200 genes in *B. subtilis* 168. In an effort to further expand our knowledge of *B. subtilis* regulation, we reconciled our model with expression data. For this process, we reconstructed the Atomic Regulons (ARs) for *B. subtilis*, which are the sets of genes that share the same “ON” and “OFF” gene expression profiles across multiple samples of experimental data. We show how ARs for *B. subtilis* are able to capture many sets of genes corresponding to regulated operons in our manually curated network. Additionally, we demonstrate how ARs can be used to help expand or validate the knowledge of the regulatory networks by looking at highly correlated genes in the ARs for which regulatory information is lacking. During this process, we were also able to infer novel stimuli for hypothetical genes by exploring the genome expression metadata relating to experimental conditions, gaining insights into novel biology.

## Introduction

Proper elucidation and characterization of gene regulatory networks has become one of the major challenges of the post-genomic era (Medini et al., 2008). Genome-wide studies of the transcriptome of the minimal organism *Mycoplasma pneumoniae* (Guell et al., 2009) and the model organism *Escherichia coli* (Cho et al., 2009) both reveal a complex regulatory architecture. This complexity can be attributed to the diversity of regulatory mechanisms in bacteria, such as transcription factors (TFs), RNA switches (Mironov et al., 2002), antisense RNA (Wagner and Simons, 1994), small RNAs (Chen and Rajewsky, 2007), or riboswitches (Nudler and Mironov, 2004). Our focus organism, *Bacillus* *subtilis*, is most commonly found in soil, and is subject to a wide variety of external environmental conditions (Illing, 2002). This reinforces the importance of understanding the regulatory mechanisms that allow the *B. subtilis* bacterium to survive and adapt to such conditions.

As a model organism, literature for *B. subtilis* regulation is extensive and several resources/databases are available. A regulatory network model for the central carbon metabolism was made available by Goelzer et al. (2008). Multiple inferred networks based on expression data have also been proposed in the literature (de Hoon et al., 2003; Steggles et al., 2007; Fadda et al., 2009). RegPrecise (Novichkov et al., 2010a), a resource for transcription factor binding site (TFBS) based network inference also provides a network for *B. subtilis* (Leyn et al., 2013). Subtiwiki (Florez et al., 2009; Michna et al., 2014) is a community collaborative resource for *B. subtilis* that includes a vast compendium of regulatory information. DBTBS (Sierro et al., 2008) is another *B. subtilis* comprehensive resource of regulatory data with promoters, TFs, TFBS, motifs and regulated operons. Our novel genome-scale reconstruction of the *B. subtilis* regulatory network integrates the previous work from the Goelzer et al. (2008) literature and the other notable resources for regulation described above (Sierro et al., 2008; Florez et al., 2009; Novichkov et al., 2010a; Mader et al., 2012).

We reconciled our new model against a large set of high-quality gene expression data (Buescher et al., 2012; Nicolas et al., 2012). For the process of reconciliation with expression data, we introduce the concept of *Atomic Regulons (ARs)*. ARs are sets of co-regulated genes that share the same “ON” and “OFF” expression profile (meaning the genes in these sets are “ON” and “OFF” in the same conditions) (submitted). Our construction of ARs began by predicting draft regulons using a combination of crude operon predictions and SEED subsystem technology (Ermolaeva et al., 2001; Overbeek et al., 2005; Price et al., 2005). We then decompose and expand these draft regulons based on consistency with expression data. This process results in the set of co-regulated gene clusters that we call AR*s*. We show how ARs can be used to help expand/validate the knowledge of the *B. subtilis* regulatory network.

## Materials and Methods

In this work, we explore the reconstruction of regulatory networks using two different approaches. In a first approach, we combine the information available in databases with notable regulatory transcriptional data for *B. subtilis* into a comprehensive manually curated regulatory network. In the second approach, we developed a methodology, dubbed “AR inference”, to infer regulatory interactions from a combination of gene expression data, predicted operons, and SEED subsystems-based annotations. For this purpose, we chose a dataset of expression data comprised of 269 samples across 104 different experimental conditions (Buescher et al., 2012; Nicolas et al., 2012). We selected this dataset for its remarkable quality, consistency, and coverage. All work to produce this dataset was conducted according to a pre-agreed Standard Operating Procedure, and only 4.4% of the known CDS in the selected strain were not expressed in any tested conditions. The whole data set is deposited in the NCBI Gene Expression Omnibus (GEO) under the accession number GSE27219. All of the different experimental conditions were performed using the BaSysBio^1^ reference strain BSB1. This strain is a tryptophan-prototrophic (trp+) derivative of the *B. subtilis* 168 trpC2 strain (Anagnostopoulos and Spizizen, 1961). Finally, to take advantage of both approaches and expand our knowledge of the *B. subtilis* regulatory network, we propose a process to reconcile their output.

We define an AR as a set of genes with identical binary (ON/OFF) expression profiles. That is, all the genes included in the same AR will always have the same state: either “all ON” or “all OFF”. This notion has meaning only in a simplified model of the cell in which genes are either ON or OFF in any condition. Thus, we must have the ability to accurately assign genes to these binary states based on their normalized expression values from a variety of experimental samples.

**FIGURE 1:**
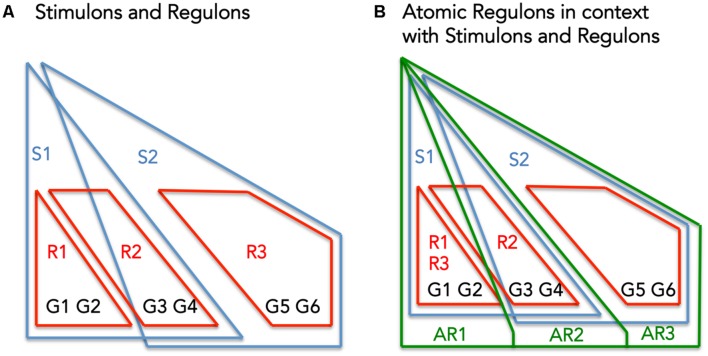
**The interplay between Stimulons, Regulons, and Atomic Regulons (ARs). (A)** The representation features six genes (G1–G6), three regulators (R1–R3), and two stimuli (S1–S2). The red lines define the regulons and the blue lines define the stimulons. **(B)** Features in addition to **(A)**: the Atomic Regulons (AR1–AR3) in the representation as sets of genes that have identical binary expression profiles.

Atomic Regulons differ subtly from existing abstractions for describing the co-regulation of genes: regulons (set of genes that respond to the same regulator), and stimulons (set of genes that respond to the same stimuli) (**Figure 1**). **Figure 1A** shows a set of six genes (G1–G6) being regulated by three regulators (R1–R3) and effected by two stimuli (S1 and S2). **Figure 1B** overlays the theoretical ARs with the previous figure.

### Atomic Regulon Curation

Genes contained within the same AR share a common expression profile; so we assume they respond to the same stimuli. Following that principle, three assertions were made for the reconciliation of our manually curated network with expression data:

(1) Each *regulon* is a subset of at least one *stimulon;*
(2) Genes often take part in multiple *stimulons*, and they will vary in whether they are induced (expression increases) or suppressed (expression decreases) in the stimulon;
(3) A set of genes that all take part in identical sets of *stimulons* with identical induction/suppression profiles comprises an AR.

**Figure 1B** demonstrates these criteria: AR1 includes genes only affected by S1, AR2 includes genes affected by S1 and S2, and AR3 includes genes only affected by S2.

A set of comparative genomics tools was used in the manual curation of the ARs. In this curation, we attempted to ensure the conservation of chromosomal gene synteny among colocalized members of an AR over phylogenetic distance. To conduct our curation, we used the “Compare regions tool” within the SEED environment (Overbeek et al., 2005). We also verified the functional annotation of AR members using comparative genomics tools like the “Compare regions tool” or the “Alignment and Tree tool” of the SEED environment. We also conducted BLASTp searches against *NCBI’s conserved domain database* (CDD) (Marchler-Bauer et al., 2015).

### The Web Resource

We developed a set of web tools to visualize all ARs we constructed for *B. subtilis*, along with the associated expression profiles and experimental metadata. The web tools are available at http://tinyurl.com/AtomicRegulons. The initial page provides the user with a list of the ARs, some of which have general descriptions.

The website can be used for other analyses of the expression data. That is, AR data presented on the web site for many different species can be used for studies outside the scope the work presented in this manuscript.

## Results and Discussion

### Draft Regulatory Network of *Bacillus subtilis* from Manual Curation

We introduce a manually constructed and curated model that describes the current state of knowledge of the transcriptional network of *B. subtilis.* The model corresponds to an updated and enlarged version of the regulation network in the central metabolism originally proposed in Goelzer et al. (2008). We have firstly extended that original network to the whole genome by including the information from the DBTBS database (Sierro et al., 2008). The DBTBS compendium of regulatory data includes promoters, TFs, TFBS, motifs, and regulated operons. The addition of the DBTBS led to a significant increase in the size of the regulatory network (**Table 1**). Additionally, we consolidated our network with all the information on regulation included in SporeWeb (Eijlander et al., 2014) and in Subtiwiki (Florez et al., 2009; Mader et al., 2012) as of March 2013. Subtiwiki is the reference community-curated resource for *B. subtilis* that was integrated into the 2012 release of DBTBS. This consolidation with Subtiwiki resulted in some revision of regulatory data included in the original network by Goelzer et al. (2008). Also, it significantly enlarged the network with respect to other microbial processes. All the above data reflect experimentally validated regulatory interactions. Additionally, RegPrecise (Novichkov et al., 2010a), a database that provides tools (Novichkov et al., 2010b) for prediction and curation of regulons, recently released their reconstruction of the regulatory network for *B. subtilis* (Leyn et al., 2013). Reconciliation with the RegPrecise inferred network resulted in the addition of a total of 39 regulators and their target gene sets to our experimentally validated network.

**Table 1:** Comparison between notable resources for *Bacillus subtilis* regulatory network modeling.

Resource	TFs	Sigma factors	RNA regulators	Effectors	Regulated genes
Goelzer et al., 2008	65	9	21	95	434
Leyn et al., 2013	129	–	33	130	1065
This work	175	19	60	169	2570

We compared the reconstruction with previously described reconstructions in the literature (**Table 1**). This comparison shows a substantial increase in network coverage from the original Goelzer et al. (2008) This increase is due in large part to an expansion of the scope of our model from the central carbon metabolism to genome-scale, as well as our effort to include most of the regulation mechanisms for *B. subtilis* that have been described in the literature to date. Our model includes 175 TF regulators, representing a wide variety of regulatory mechanisms: TFs conditioned by metabolites, accessory proteins, phosphorylated proteins, and stress factors. Sigma factor regulation was also included as it plays a role in governing many major cell functions such as sporulation (sigE, sigF, sigG, and sigK), regulation of flagella, motility, and chemotaxis (sigD), cell wall surface properties, and stress (sigX, sigW, and sigV). Elements relating to anti-sense RNA, riboswitches, RNA switches, RNA antiterminators, and small regulatory RNAs compose the 60 RNA regulators described in our network.

The increase in the number of regulators in our model led to a corresponding increase in effectors. We divide our effectors into two categories: biochemical (involving metabolites), and environmental effectors (e.g., DNA damage and heat shock). The 275 regulators in our model are linked to a set of regulons comprised of ∼2500 genes. However, notably, the detailed regulatory mechanisms associated with some of the regulons in our model, particularly in cases of sigma factor and RNA regulation, remain unclear, or unknown. All details related to the regulatory model, including data sources are provided in **Supplementary Tables S1** and **S2**. An online version is also available at http://modelseed.org/projects/regulons.

**FIGURE 2:**
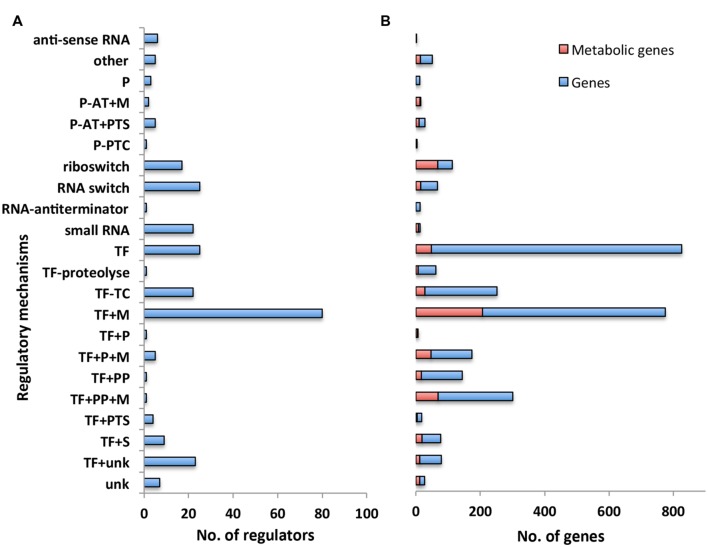
**Overview of regulatory network categorized by regulatory mechanism. (A)** Number of regulators with respect to their regulatory mechanisms. Each bar corresponds to the number of regulators having the same type of regulatory mechanism. **(B)** Number of genes with respect to the regulatory mechanisms controlling their expression. Each bar refers to the number of genes that are controlled by a regulator having the same type of regulatory mechanism. Number of genes involved in the metabolism is highlighted in red. Abbreviations: P (Accessory protein involved in regulation); P-AT+M (Protein – transcriptional antiterminator conditioned by a metabolite); P-AT+PTS (Protein – transcriptional antiterminator conditioned by a PTS phosphorylation); P-PTC (Protein post-transcriptional control); TF (Transcription factor); TF–TC (Two-component response regulator); TF+M (Transcription factor conditioned by a metabolite); TF+P (Transcription factor + accessory protein); TF+P+M (Transcription factor + accessory protein associated to a metabolite); TF+PP (Transcription Factor + phosphorylated protein); TF+PP+M (Transcription factor + phosphorylated protein + metabolite); TF+PTS (Transcription factor + PTS phosphorylation); TF+S [Transcription factor + stress (DNA alteration/TF alteration)]; TF+unk (Transcription factor conditioned by an unknown mechanism/protein/metabolite).

We first classified the 275 regulators in our model with respect to their associated regulatory mechanism (**Figure 2A**). Some regulatory mechanisms were not completely known. We thus introduced additional categories such as TFs conditioned by an unknown regulatory mechanism (“TF+unk”) to indicate the partial level of knowledge on these mechanisms (see **Supplementary Table S1** for the full list and description of categories). 40% of the regulators have a mechanism that directly responds to a metabolic signal (categories TFs conditioned by a metabolite (“TF+M”), TFs + phosphorylated protein + metabolite (“TF + PP + M”), TFs + accessory protein associated to a metabolite (“TF + P + M”), Protein – transcriptional antiterminator conditioned by a PTS phosphorylation (“P-AT + PTS”), Protein – transcriptional antiterminator conditioned by a metabolite (“P-AT + M”), riboswitch). In particular, the main regulatory mechanism (“TF + M”, associated with 25% of the regulators) involves a TFs having one (or several) metabolite(s) as direct effectors. These results emphasized the work of Goelzer et al. (2008) that originally pointed out the weight of metabolism in the genetic regulation of metabolic pathways. Metabolites are strongly involved in the activation of regulators at genome-scale.

We then examined the number of genes responding to the regulators associated with each distinct regulatory mechanism (**Figure 2B**). In agreement with the experimental results of (Nicolas et al., 2012), we found the largest number of genes were associated with mechanisms involving a sigma factor (Sigma factor regulatory mechanism is not picture in **Figure 2B** due to the disproportionally large number of genes associated with this mechanism, all details are available in **Supplementary Tables S1** and **S2**). All together, these mechanisms were associated with 40% of the genes included in our model. Moreover, 26% of the genes in our model were associated with regulatory mechanisms that respond to a metabolic signal (categories “TF+M”, “TF+PP+M”, “TF+P+M”, “P-AT+PTS”, “P-AT+M”, “riboswitch”); this set of genes includes 45% of the genes involved in the metabolic pathways of *B. subtilis* (Henry et al., 2009) (**Figure 2B** highlighted in red), in agreement with results from previous work (Goelzer et al., 2008). This confirms the key role of metabolites in the regulation of metabolic pathways.

In addition to sigma factors, the other main regulatory mechanisms, representing ∼29% of the reported regulatory interactions, were TFs alone (“TF”) and the TFs having one (or several) metabolites as direct effectors (“TF+M”) (**Figure 2**). Most of the regulators of the category “TF+M” correspond to local regulators, following the definition of Goelzer et al. (2008): “the local regulator of a metabolic pathway guarantees the activation or the inhibition of genes as a function of an intermediate metabolite of the metabolic pathway”. Interestingly, the complex regulatory mechanisms involving several entities, like a TFs and a phosphorylated protein with or without a metabolite (“TF+PP” and “TF+PP+M”) had only a single pleiotropic regulator with multiple target genes. These regulators are Spo0A and CcpA and are involved, respectively, in the cell fate decision (Lopez and Kolter, 2010) and in the management of carbon sources (Fujita, 2009). Increasing the complexity of the mechanism of regulation may reflect the necessity for the cell to integrate different signals or to have a large range of modulation in the gene expression.

### Atomic Regulons for *Bacillus subtilis*

**FIGURE 3:**
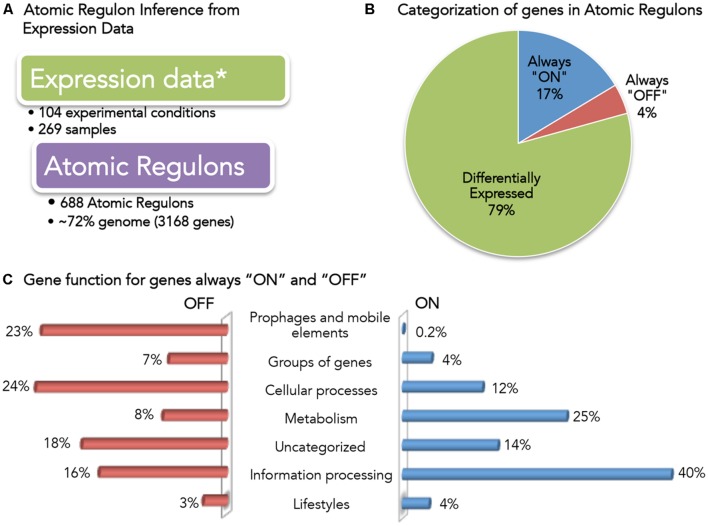
**Overview of *Bacillus subtilis* Atomic Regulons (ARs). (A)** Expression data used for AR inference in *B. subtilis* (Buescher et al., 2012; Nicolas et al., 2012). **(B)** Categorization of genes in ARs. Genes have been categorized based on the expression profile as always “ON”, always “OFF” and differentially expressed. **(C)** Gene function for genes always “ON” and “OFF”. *B. subtilis* 168 genes are classified in SubtiWiki (Florez et al., 2009; Mader et al., 2012) into 6 categories cellular function; here we show the fraction of genes in the “always ON” regulon (right) and the “always OFF” regulon (left) that occur in each category.

With the initial reconstruction of our new regulatory model of *B. subtilis* complete, we next applied gene expression data to reconcile and validate our model. This process began with a survey of the data present in current expression databases. As of May 2015, there were ∼1750 datasets in GEO (Edgar et al., 2002) related to *B. subtilis* strains. However, the utilization of all of these data in our model validation posed a challenge, as protocols vary from lab to lab, and various expression platforms can produce results that are difficult to merge. Thus, we selected a single dataset, comprised of 269 samples collected from 104 different growth conditions (Buescher et al., 2012; Nicolas et al., 2012). We used this data with our new algorithm to infer a set of ARs for the *B. subtilis 168* genome (**Figure 3** and **Supplementary Table S3**).

A total of 688 ARs were computed comprising 3168 genes (∼72% of the genome) (**Figure 3A**). We categorized these ARs according to their expression profile (**Figure 3B**): only 4% (137) of the genes were always OFF in all conditions, while 17% (523) of the genes were ON in all conditions. This result was consistent with the claim by the authors of the study that 95% of the genes in *B. subtilis* had been expressed in at least one condition. We explored the functions associated with the genes that we found to be always ON or always OFF (**Figure 3C**).

Forty percent of the always-ON genes (211) are categorized as information processing, which encompasses: RNA synthesis and degradation (transcription); protein folding, modification and degradation and translation; and, DNA replication. 25% (129) of the always-ON genes were metabolic, including central carbon, nucleotide, and lipid metabolism. Finally, 12% (63) of the always-ON genes were associated with cellular process, including cell wall biosynthesis, cell division, transporters and homeostasis.

The small set of genes (137) found to be OFF in all conditions is comprised of genes across a diverse set of functions. To verify that no gene found to be OFF in all conditions was an essential gene, we compared the set with a list of *B. subtilis* essential genes (Kobayashi et al., 2003; Commichau et al., 2013). No essential *B. subtilis* genes were found to be OFF in all conditions.

### Reconciling Expression Data with the Draft Regulatory Network for *Bacillus subtilis*

**FIGURE 4:**
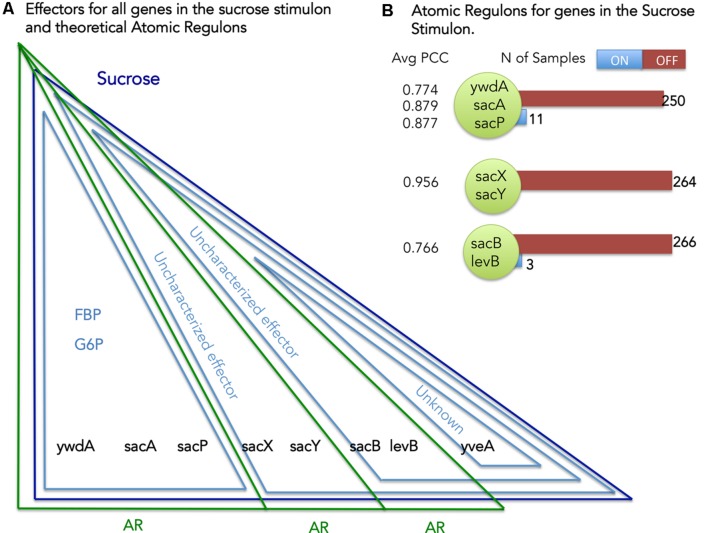
**Atomic Regulons (ARs) for the sucrose stimulon. (A)** Effectors for all genes in the sucrose stimulon and theoretical ARs. Eight genes compose the sucrose stimulon (dark blue triangle). Fructose-biphospshate (FBP), Glucose-6-phosphate and uncharacterized effectors are also effectors (light blue triangles). The theoretical ARs are represented in green triangles. **(B)** ARs inferred for the sucrose stimulon. The ARs that were inferred are shown with the average Pearson correlation coefficient (PCC) and with a listing of the members of the AR. Number of samples ON and OFF for each AR is also shown.

Our definition of AR states that genes contained within the same AR must respond to the same set of stimuli (**Figure 1**). We can use this principle to identify and reconcile inconsistencies that exist between the stimuli mapped to the genes in our *B. subtilis* model and the set of genes comprising each AR. Considering sucrose as an example (**Figure 4**) we can explore the set of ARs computed for the genes comprising the Sucrose stimulon. We have eight genes in the Sucrose stimulon that respond to four distinct combinations of stimuli: (i) *ywdA*, *sacA*, and *sacP* all respond to both fructose-biphosphate and glucose-6-phosphate (Debarbouille et al., 1990); (ii) sacX and sacY respond to an uncharacterized stimulus (Tortosa and Le Coq, 1995); (iii) sacB and levB respond to two uncharacterized stimuli (Tortosa and Le Coq, 1995); and (iv) yveA shares the same uncharacterized stimuli as the previous genes, plus another uncharacterized stimulus (Pereira et al., 2001).

Our AR inference algorithm divided the sucrose stimulon into three ARs (**Table 2** and **Figure 4**): (i) AR #376 containing *ywdA*, *sacA*, and *sacP*, (ii) AR #254 containing sacX and sacY; and (iii) AR #625 containing sacB and levB. yveA was not placed into an AR since the methodology requires two or more genes for placement in AR. The capacity of our AR inference methodology to divide these genes into separate ARs that conform perfectly with their distinctive stimuli demonstrates the robustness of the approach. Two of these ARs, #254 and #625, respond to uncharacterized effectors in our model (**Figure 4A**), and these ARs are active in no more than five and three experiments in our expression dataset respectively (**Figure 4B**). For AR 625 we checked the experiments in which the genes were ON (**Table 3**). Genes in AR 625 were found to be “ON” in experiments that tested gene expression at regular intervals after sporulation was induced. A detailed description of the study can be found by looking at the associated study number (in this case “study0003”^2^). Sporulation was induced with the use of CH medium (Sterlini and Mandelstam, 1969), and cells were harvested at hourly intervals, with the genes in our ARs being ON in the late intervals of the study. This fact tells us that our uncharacterized effector can be related with sporulation and that a compound in the CH medium can be a candidate for the uncharacterized effector.

**Table 2:** Sucrose stimulon represented in the Atomic Regulon (AR) web analysis resource.

AR number^∗^	Gene name/BSU	Stimuli
254	sacX, BSU38410	Uncharacterized, Sucrose
254	sacY, BSU38420	Uncharacterized, Sucrose
376	ywdA, BSU38030	D-fructose-1,6-bisphosphate, Glucose-6-Phosphate, Sucrose
376	sacA, BSU38040	D-fructose-1,6-bisphosphate, Glucose-6-Phosphate, Sucrose
376	sacP, BSU38050	D-fructose-1,6-bisphosphate, Glucose-6-Phosphate, Sucrose
625	sacB, BSU34450	Uncharacterized, uncharacterized, Sucrose
625	levB, BSU34460	Uncharacterized, uncharacterized, Sucrose

*^∗^AR number arbitrarily assigned by the inference algorithm (the ARs described **Figure 4** were assigned AR numbers 254, 376, and 625)*.

**Table 3:** Experiments in which Atomic Regulon 625 was found to be “ON”.

Study	Sample	Study explanation
study0003	S6/t_2_hyb42359702	Tested gene expression at regular intervals after sporulation was induced
study0003	S6_2_hyb29634602	Tested gene expression at regular intervals after sporulation was induced
study0003	S8_5_hyb43271102	Tested gene expression at regular intervals after sporulation was induced

To assess the consistency of all ARs when compared with the stimuli in the regulatory network, we organized all computed ARs into four categories: consistent; consistent with missing stimuli; inconsistent; and empty (counts in **Table 4**). The *consistent* category comprises ARs with members that have the same stimuli/effectors in the regulatory network. The *consistent with missing stimuli* category comprises cases in which a member of the AR has the same stimuli/effectors, while other members have no described stimuli in the regulatory network. The *inconsistent* category displays ARs with members that have different stimuli associated in the regulatory network. ARs with no stimuli/effectors described in the regulatory network were categorized as *empty*. We used these results to further curate and refine our model, and we report the results of this analysis prior to curation (V1) and after curation (V2). Here, we highlight some of the considerations we made during the process of manual curation, describing our analysis of several ARs as examples.

**Table 4:** Consistency of Atomic Regulons (ARs) with the regulatory network.

Classification	V1	V2
Consistent (+)	151	174
Consistent with missing stimuli (±)	74	45
Inconsistent (^∗^)	48	32
Empty (-)	415	425
Total	688	676

*Reflects the consistency of the original ARs (V1) and the curated ARs (V2). (+) All ARs members have the same stimuli/effectors in the regulatory network. (±) Some members of the AR have the same stimuli/effectors while other members have no described stimuli in the regulatory network. (^∗^) ARs members have different stimuli associated in the regulatory network. (-) No stimuli described in the regulatory network*.

In some cases, our curation of the *inconsistent* ARs led to the removal of genes from the AR, which inconsistencies proved to be biologically meaningful. AR #56 is one such example (**Table 5**). All members of AR #56, except *zur*, respond to hydrogen peroxide as a stimuli; and upon further inspection, we note that AR #56 corresponds almost perfectly with the *hemAXCDBL* operon (Hansson et al., 1991), which has been found to be regulated by PerR and is induced by hydrogen peroxide (Fuangthong et al., 2002). The inconsistent gene in AR #56, Zur, is a PerR paralog (Fuangthong and Helmann, 2003) involved in regulation of the zinc homeostasis as the zinc uptake repressor (Gaballa et al., 2002). *zur* was removed from AR #56, making this AR consistent according to our previously described crtieria. This finding also highlighted the fact that many of our ARs include regulatory genes in addition to regulatory gene target, which is undesirable for regulons being used within a regulatory network model. We thus used the information from our manually curated network to remove all known regulatory proteins from the ARs.

**Table 5:** Atomic Regulon 56.

Gene name / BSU	Stimuli	Average PCC
zur, BSU25100		0.664
hemL, BSU28120	Hydrogen peroxide	0.885
hemB, BSU28130	Hydrogen peroxide	0.900
hemD, BSU28140	Hydrogen peroxide	0.905
hemC, BSU28150	Hydrogen peroxide	0.906
hemX, BSU28160	Hydrogen peroxide	0.893
hemA, BSU28170	Hydrogen peroxide	0.804

*Stimuli and average Pearson correlation coefficient (PCC) among members of the AR are shown for each gene (Gene names and *B. subtilis* specific BSU numbers provided) in the AR*.

In other cases, our curation of the inconsistent AR aided us in identifying gene-stimuli interactions that were missing from our initial model. An example of this is AR #612, which is comprised of 10 genes having functions associated with heme/iron transport (**Table 6**). From our regulatory network model, we have “Iron” associated with 8 out of the 10 AR members. A survey for *yetG* (now *hmoA*) revealed that the gene has been recently characterized to encode a heme-degrading monooxygenase (Gaballa and Helmann, 2011). *hmoA* has also been shown to be regulated by *Fur*, the same regulator as the other members of AR #612. There was no information on iron as stimuli for the *yetH* gene product, and it had the lowest average PCC among all members of AR #612. Despite those two facts, *yetH* should remain part of the AR #612 because the gene product is a member of the Glyoxalase/bleomycin resistance protein family and is known to be a metalloprotein. We verified this by using BLASTp against *NCBI’s CDD*. This insight has been reflected in our *B. subtilis* SEED annotation by changing the functional role to “Glyoxalase/bleomycin resistance family metalloprotein”. We suggest the expansion of regulatory information for *hmoA* and *yetH* to be consistent within AR #612 with the addition of iron as stimuli and *Fur* as regulator (the latest release of Subtiwiki has already implemented one of the changes for *hmoA*).

**Table 6:** Atomic Regulon 612.

Gene name / BSU	Stimuli	Average PCC
yclN, BSU03800	Iron	0.804
yclO, BSU03810	Iron	0.807
yclP, BSU03820	Iron	0.815
yclQ, BSU03830	Iron	0.806
yetG, BSU07150		0.716
yetH, BSU07160		0.605
yfmF, BSU07490	Iron	0.621
yfmE, BSU07500	Iron	0.702
yfmD, BSU07510	Iron	0.769
yfmC, BSU07520	Iron	0.764
yhfQ, BSU10330	Iron	0.779

*Stimuli and average PCC among members of the AR are shown for each gene (Gene names and *B. subtilis* specific BSU numbers provided) in the AR*.

Other inconsistencies involved genes in ARs where all members of the AR did not share the same set of stimuli. An example of this case is AR #332 (**Table 7**). Three out of four members of the AR #332 share the same effectors. These three members (*treP*, *treA*, and *treR*) were found to comprise the tre operon (Schock and Dahl, 1996). TreR is a transcriptional repressor, involved in the regulation of trehalose utilization and it is inhibited by trehalose-6-phosphate (Burklen et al., 1998). The additional stimuli, D-fructose-1,6-bisphosphate and Glucose-6-Phosphate, relate to the activity of the carbon catabolite repression global regulator CcpA (Henkin, 1996). The fourth member of the AR, *yfkO*, has been described in the literature as a nitroreductase (Prosser et al., 2010). Upon inspection of this region of the chromosome, we found *yfkO* up-stream of the transcriptional regulator TreR, and not a member of the tre operon/TreR regulon. Due to this analysis we removed *yfkO* from AR 332. As noted before, we also removed TreR as the protein imposing the regulatory activity. This curated AR was classified as “Trehalose Utilization”.

**Table 7:** Atomic Regulon 332.

Gene name / BSU	Stimuli	Average PCC	Function
treP, BSU07800	D-fructose-1, 6-bisphosphate, Glucose-6-Phosphate, phosphate, D-trehalose-6-phosphate	0.807	PTS system, trehalose-specific IIB component (EC 2.7.1.69)
treA, BSU07810	D-fructose-1, 6-bisphosphate, Glucose-6-Phosphate, phosphate, D-trehalose-6-phosphate	0.814	Trehalose-6-phosphate hydrolase (EC 3.2.1.93)
treR, BSU07820	D-fructose-1, 6-bisphosphate, Glucose-6-Phosphate, phosphate, D-trehalose-6-phosphate	0.734	Trehalose operon transcriptional repressor
yfkO, BSU07830	Disulfide_stress_conditions	0.706	Oxygen-insensitive NAD(P)H nitroreductase (EC 1.-.-.-)

*Stimuli, average PCC among members of the AR and functional role is shown for each gene (Gene names and *B. subtilis* specific BSU numbers provided) in the AR*.

Four hundred and fifteen ARs were found to have no associated stimuli in the regulatory network. Previously, we attempted to use details in the gene expression experiments to aid in the characterization of unknown effectors (**Figure 4**). We applied the same approach to ARs for which there are no effectors in the regulatory network, such as AR #651 (**Table 8**). In addition to having no associated regulator or stimuli in the network, all genes in AR #651 have unknown functions. The members of AR #651 show a high average PCC and are only “ON” in a very small number of samples. In the experiment that activated AR #651, cells were grown in LB medium at 37°C with vigorous shaking. During exponential growth (O.D.600 ∼0.25), the cell culture was divided into two subcultures: one subculture acted as control [no mitomycin C, M0], while mitomycin was added to the second subculture at a concentration of 40 ng/mL [mitomycin, M40]. Samples were harvested at 0, 45 and 90 min after mitomycin addition [t0, t45, and t90]. Addition of mitomycin C promoted prophage induction.

**Table 8:** Atomic Regulon 651.

Gene name / BSU	Stimuli	Average PCC	Function	OFF	ON
yosW, BSU19980	–	0.906	unknown	261	4
yosV, BSU19990	–	0.948	unknown	261	4
yojW, BSU19999	–	0.941	unknown	261	4

*Stimuli, average PCC among members of the AR and functional role are shown for each gene (Gene names and *B. subtilis* specific BSU numbers provided) in the AR*.

We used our web tools^3^ to identify all the genes that changed state between our control sample (grown in LB media only) and the sample grown with mitomycin C^4^. In the results, we see several AR associated with prophages being ON in the experiment wherein mitomycin C was added. Mitomycin C serves to stimulate the expression of these specific genes, leading to its addition as a stimulus for these ARs.

The “case studies” presented before were part a larger manual curation effort. All changes made to ARs during the curation process can be found in **Supplementary Table S4**. In **Table 4** we can see the impact of the curation process (V2) across our four categories of AR consistency. We see a decrease in ARs categorized as *inconsistent* and *consistent with missing stimuli*. This led to a subsequent increase in the number of *consistent* ARs. The improved set (**Supplementary Tables S5** and **S6**) contains new versions of ARs and the Regulatory network that reflect all the changes made during the manual curation process.

## Conclusion

We introduce a new, expanded regulatory network for *B. subtilis*, compiling information from multiple notable sources of gene regulatory data as well as our own inferences. We show that our reconstruction is more comprehensive than other previous versions found in the literature. This new network is a valuable resource of known regulatory interactions occurring within *B. subtilis 168*, and it may be readily integrated with existing genome-scale metabolic models of *B. subtilis* (Henry et al., 2009; Tanaka et al., 2013) to improve model predictions by accounting for regulatory interactions (Covert and Palsson, 2002; Herrgard et al., 2004). We demonstrate through our analysis of the model the prevalence of regulators responding to small molecule effectors. We also show the prevalence of sigma-factor regulation in *B. subtilis*, in terms of number of impacted genes. Finally, we explore how the most complex regulation is used to trigger major system responses to a wide range of environmental conditions.

Additionally, we introduce the use of AR*s* to reconcile our proposed network with available gene expression data. We show how this methodology is able to fill gaps and correct inconsistencies in the regulatory network. The reconciliation process allowed us to expand our knowledge of *B. subtilis* regulation by adding new stimuli to genes and regulons, by adjusting stimuli on existing genes, and by adjust regulons based on inconsistent expression patterns. We were also able to provide clues for putative gene function assignments for genes with unknown functions.

All AR data were integrated into the PubSEED^5^, and they can be used as part of the annotation curation tools available in that framework. A web resource was also created showing the relationship between the ARs and the expression data used for their computation in *B. subtilis*. This resource is available for the public and can be used to conduct analysis of the ARs and expression data sets beyond the objectives of the work described in this manuscript.

During the reconciliation process, we were able to match many ARs to the same regulatory mechanisms we found in our manually curated network. This fact highlights the convenience of using ARs to study the regulatory network of an organism without a huge effort put into initial manual curation.

## Author Contributions

JF, AG, and VF contributed to the regulatory network reconstruction manual curation efforts from notable *Bacilus subtilis* regulation resources. The inference of Atomic Regulons was performed by JF, RO, and RT. JF, VV, and RO performed the reconciliation of the regulatory network with the gene expression data. JF, MR, IR, and CH contributed to the analysis of the new reconciled regulatory network. RO and NC contributed to the web resources introduced in the manuscript. All authors contributed to data analysis and manuscript preparation. CH conceived of the project and oversaw work.

## Conflict of Interest Statement

The authors declare that the research was conducted in the absence of any commercial or financial relationships that could be construed as a potential conflict of interest.

## Footnotes

1 http://www.basysbio.eu/

2 http://tinyurl.com/ARStudies

3 http://tinyurl.com/ARStudies

4 http://tinyurl.com/LBvsMitomycin

5 http://pubseed.theseed.org/

## Supplementary Material

TABLE S1 **Manually curated regulatory network for *Bacillus subtilis***.

TABLE S2 **Regulators described in the regulatory network**.

TABLE S3 **Atomic Regulons for *B. subtilis***.

TABLE S4 **Curation of Atomic Regulons**.

TABLE S5 **Atomic Regulons or *B. subtilis* (version 2)**.

TABLE S6 **Manually curated regulatory network for *B. subtilis* (version 2)**.

## References

[B1] Anagnostopoulos C. Spizizen J. (1961). Requirements for transformation in *Bacillus subtilis*. *J. Bacteriol.* 81 741–746. 16561900 10.1128/jb.81.5.741-746.1961 PMC279084

[B2] Buescher J. M. Liebermeister W. Jules M. Uhr M. Muntel J. Botella E. (2012). Global network reorganization during dynamic adaptations of *Bacillus subtilis* metabolism. *Science* 335 1099–1103. 10.1126/science.1206871 22383848

[B3] Burklen L. Schock F. Dahl M. K. (1998). Molecular analysis of the interaction between the *Bacillus subtilis* trehalose repressor TreR and the tre operator. *Mol. Gen. Genet.* 260 48–55. 10.1007/s004380050869 9829827

[B4] Chen K. Rajewsky N. (2007). The evolution of gene regulation by transcription factors and microRNAs. *Nat. Rev. Genet.* 8 93–103. 10.1038/nrg1990 17230196

[B5] Cho B. K. Zengler K. Qiu Y. Park Y. S. Knight E. M. Barrett C. L. (2009). The transcription unit architecture of the *Escherichia coli* genome. *Nat. Biotechnol.* 27 1043–1049. 10.1038/nbt.1582 19881496 PMC3832199

[B6] Commichau F. M. Pietack N. Stulke J. (2013). Essential genes in *Bacillus subtilis*: a re-evaluation after ten years. *Mol. Biosyst.* 9 1068–1075. 10.1039/c3mb25595f 23420519

[B7] Covert M. W. Palsson B. O. (2002). Transcriptional regulation in constraints-based metabolic models of *Escherichia coli*. *J. Biol. Chem.* 277 28058–28064. 10.1074/jbc.M201691200 12006566

[B8] de Hoon M. J. Imoto S. Kobayashi K. Ogasawara N. Miyano S. (2003). Inferring gene regulatory networks from time-ordered gene expression data of *Bacillus subtilis* using differential equations. *Pac. Symp. Biocomput.* 8 17–28. 12603014

[B9] Debarbouille M. Arnaud M. Fouet A. Klier A. Rapoport G. (1990). The sacT gene regulating the sacPA operon in *Bacillus subtilis* shares strong homology with transcriptional antiterminators. *J. Bacteriol.* 172 3966–3973. 2163394 10.1128/jb.172.7.3966-3973.1990 PMC213381

[B10] Edgar R. Domrachev M. Lash A. E. (2002). Gene expression omnibus: NCBI gene expression and hybridization array data repository. *Nucleic Acids Res.* 30 207–210. 10.1093/nar/30.1.207 11752295 PMC99122

[B11] Eijlander R. T. de Jong A. Krawczyk A. O. Holsappel S. Kuipers O. P. (2014). SporeWeb: an interactive journey through the complete sporulation cycle of *Bacillus subtilis*. *Nucleic Acids Res.* 42 D685–D691. 10.1093/nar/gkt1007 24170806 PMC3964945

[B12] Ermolaeva M. D. White O. Salzberg S. L. (2001). Prediction of operons in microbial genomes. *Nucleic Acids Res.* 29 1216–1221. 10.1093/nar/29.5.1216 11222772 PMC29727

[B13] Fadda A. Fierro A. C. Lemmens K. Monsieurs P. Engelen K. Marchal K. (2009). Inferring the transcriptional network of *Bacillus subtilis*. *Mol. Biosyst.* 5 1840–1852. 10.1039/b907310h 20023724

[B14] Florez L. A. Roppel S. F. Schmeisky A. G. Lammers C. R. Stulke J. (2009). A community-curated consensual annotation that is continuously updated: the *Bacillus subtilis* centred wiki SubtiWiki. *Database (Oxford)* 2009:ba012 10.1093/database/bap012 PMC2790307 20157485

[B15] Fuangthong M. Helmann J. D. (2003). Recognition of DNA by three ferric uptake regulator (Fur) homologs in *Bacillus subtilis*. *J. Bacteriol.* 185 6348–6357. 10.1128/JB.185.21.6348-6357.2003 14563870 PMC219410

[B16] Fuangthong M. Herbig A. F. Bsat N. Helmann J. D. (2002). Regulation of the Bacillus *subtilis* fur and perR genes by PerR: not all members of the PerR regulon are peroxide inducible. *J. Bacteriol.* 184 3276–3286. 10.1128/JB.184.12.3276-3286.2002 12029044 PMC135084

[B17] Fujita Y. (2009). Carbon catabolite control of the metabolic network in *Bacillus subtilis*. *Biosci. Biotechnol. Biochem.* 73 245–259. 10.1271/bbb.80479 19202299

[B18] Gaballa A. Helmann J. D. (2011). *Bacillus subtilis* Fur represses one of two paralogous haem-degrading monooxygenases. *Microbiology* 157 3221–3231. 10.1099/mic.0.053579-0 21873409 PMC3352277

[B19] Gaballa A. Wang T. Ye R. W. Helmann J. D. (2002). Functional analysis of the *Bacillus subtilis* Zur regulon. *J. Bacteriol.* 184 6508–6514. 10.1128/JB.184.23.6508-6514.2002 12426338 PMC135443

[B20] Goelzer A. Bekkal Brikci F. Martin-Verstraete I. Noirot P. Bessieres P. Aymerich S. (2008). Reconstruction and analysis of the genetic and metabolic regulatory networks of the central metabolism of *Bacillus subtilis*. *BMC Syst. Biol.* 2:20 10.1186/1752-0509-2-20 PMC2311275 18302748

[B21] Guell M. van Noort V. Yus E. Chen W. H. Leigh-Bell J. Michalodimitrakis K. (2009). Transcriptome complexity in a genome-reduced bacterium. *Science* 326 1268–1271. 10.1126/science.1176951 19965477

[B22] Hansson M. Rutberg L. Schroder I. Hederstedt L. (1991). The *Bacillus subtilis* hemAXCDBL gene cluster, which encodes enzymes of the biosynthetic pathway from glutamate to uroporphyrinogen III. *J. Bacteriol.* 173 2590–2599. 1672867 10.1128/jb.173.8.2590-2599.1991 PMC207825

[B23] Henkin T. M. (1996). The role of CcpA transcriptional regulator in carbon metabolism in *Bacillus subtilis*. *FEMS Microbiol. Lett.* 135 9–15. 10.1111/j.1574-6968.1996.tb07959.x 8598282

[B24] Henry C. S. Zinner J. F. Cohoon M. P. Stevens R. L. (2009). iBsu1103: a new genome-scale metabolic model of *Bacillus subtilis* based on SEED annotations. *Genome Biol.* 10:R69 10.1186/gb-2009-10-6-r69 PMC2718503 19555510

[B25] Herrgard M. J. Covert M. W. Palsson B. O. (2004). Reconstruction of microbial transcriptional regulatory networks. *Curr. Opin. Biotechnol.* 15 70–77. 10.1016/j.copbio.2003.11.002 15102470

[B26] Illing N. (2002). *Bacillus subtilis* and its closest relatives: from genes to cells. *Nature* 415 263–264. 10.1038/415263b

[B27] Kobayashi K. Ehrlich S. D. Albertini A. Amati G. Andersen K. K. Arnaud M. (2003). Essential *Bacillus subtilis* genes. *Proc. Natl. Acad. Sci. U.S.A.* 100 4678–4683. 10.1073/pnas.0730515100 12682299 PMC153615

[B28] Leyn S. A. Kazanov M. D. Sernova N. V. Ermakova E. O. Novichkov P. S. Rodionov D. A. (2013). Genomic reconstruction of the transcriptional regulatory network in *Bacillus subtilis*. *J. Bacteriol.* 195 2463–2473. 10.1128/JB.00140-13 23504016 PMC3676070

[B29] Lopez D. Kolter R. (2010). Extracellular signals that define distinct and coexisting cell fates in *Bacillus subtilis*. *FEMS Microbiol. Rev.* 34 134–149. 10.1111/j.1574-6976.2009.00199.x 20030732

[B30] Mader U. Schmeisky A. G. Florez L. A. Stulke J. (2012). SubtiWiki–a comprehensive community resource for the model organism *Bacillus subtilis*. *Nucleic Acids Res.* 40 D1278–D1287. 10.1093/nar/gkr923 22096228 PMC3245094

[B31] Marchler-Bauer A. Derbyshire M. K. Gonzales N. R. Lu S. Chitsaz F. Geer L. Y. (2015). CDD: NCBI’s conserved domain database. *Nucleic Acids Res.* 43 D222–D226. 10.1093/nar/gku1221 25414356 PMC4383992

[B32] Medini D. Serruto D. Parkhill J. Relman D. A. Donati C. Moxon R. (2008). Microbiology in the post-genomic era. *Nat. Rev. Microbiol.* 6 419–430. 10.1038/nrmicro1901 18475305

[B33] Michna R. H. Commichau F. M. Todter D. Zschiedrich C. P. Stulke J. (2014). SubtiWiki-a database for the model organism *Bacillus subtilis* that links pathway, interaction and expression information. *Nucleic Acids Res.* 42 D692–D698. 10.1093/nar/gkt1002 24178028 PMC3965029

[B34] Mironov A. S. Gusarov I. Rafikov R. Lopez L. E. Shatalin K. Kreneva R. A. (2002). Sensing small molecules by nascent RNA: a mechanism to control transcription in bacteria. *Cell* 111 747–756. 10.1016/S0092-8674(02)01134-0 12464185

[B35] Nicolas P. Mader U. Dervyn E. Rochat T. Leduc A. Pigeonneau N. (2012). Condition-dependent transcriptome reveals high-level regulatory architecture in *Bacillus subtilis*. *Science* 335 1103–1106. 10.1126/science.1206848 22383849

[B36] Novichkov P. S. Laikova O. N. Novichkova E. S. Gelfand M. S. Arkin A. P. Dubchak I. (2010a). RegPrecise: a database of curated genomic inferences of transcriptional regulatory interactions in prokaryotes. *Nucleic Acids Res.* 38 D111–D118. 10.1093/nar/gkp894 19884135 PMC2808921

[B37] Novichkov P. S. Rodionov D. A. Stavrovskaya E. D. Novichkova E. S. Kazakov A. E. Gelfand M. S. (2010b). RegPredict: an integrated system for regulon inference in prokaryotes by comparative genomics approach. *Nucleic Acids Res.* 38 W299–W307. 10.1093/nar/gkq531 20542910 PMC2896116

[B38] Nudler E. Mironov A. S. (2004). The riboswitch control of bacterial metabolism. *Trends Biochem. Sci.* 29 11–17. 10.1016/j.tibs.2003.11.004 14729327

[B39] Overbeek R. Begley T. Butler R. M. Choudhuri J. V. Chuang H. Y. Cohoon M. (2005). The subsystems approach to genome annotation and its use in the project to annotate 1000 genomes. *Nucleic Acids Res.* 33 5691–5702. 10.1093/nar/gki866 16214803 PMC1251668

[B40] Pereira Y. Petit-Glatron M. F. Chambert R. (2001). yveB, Encoding endolevanase LevB, is part of the sacB-yveB-yveA levansucrase tricistronic operon in *Bacillus subtilis*. *Microbiology* 147 3413–3419. 10.1099/00221287-147-12-3413 11739774

[B41] Price M. N. Huang K. H. Alm E. J. Arkin A. P. (2005). A novel method for accurate operon predictions in all sequenced prokaryotes. *Nucleic Acids Res.* 33 880–892. 10.1093/nar/gki232 15701760 PMC549399

[B42] Prosser G. A. Patterson A. V. Ackerley D. F. (2010). uvrB gene deletion enhances SOS chromotest sensitivity for nitroreductases that preferentially generate the 4-hydroxylamine metabolite of the anti-cancer prodrug CB1954. *J. Biotechnol.* 150 190–194. 10.1016/j.jbiotec.2010.08.007 20727918

[B43] Schock F. Dahl M. K. (1996). Expression of the tre operon of *Bacillus subtilis* 168 is regulated by the repressor TreR. *J. Bacteriol.* 178 4576–4581. 8755887 10.1128/jb.178.15.4576-4581.1996 PMC178226

[B44] Sierro N. Makita Y. de Hoon M. Nakai K. (2008). DBTBS: a database of transcriptional regulation in *Bacillus subtilis* containing upstream intergenic conservation information. *Nucleic Acids Res.* 36 D93–D96. 10.1093/nar/gkm910 17962296 PMC2247474

[B45] Steggles L. J. Banks R. Shaw O. Wipat A. (2007). Qualitatively modelling and analysing genetic regulatory networks: a Petri net approach. *Bioinformatics* 23 336–343. 10.1093/bioinformatics/btl596 17121774

[B46] Sterlini J. M. Mandelstam J. (1969). Commitment to sporulation in Bacillus *subtilis* and its relationship to development of actinomycin resistance. *Biochem. J.* 113 29–37. 10.1042/bj1130029 4185146 PMC1184601

[B47] Tanaka K. Henry C. S. Zinner J. F. Jolivet E. Cohoon M. P. Xia F. (2013). Building the repertoire of dispensable chromosome regions in *Bacillus subtilis* entails major refinement of cognate large-scale metabolic model. *Nucleic Acids Res.* 41 687–699. 10.1093/nar/gks963 23109554 PMC3592452

[B48] Tortosa P. Le Coq D. (1995). A ribonucleic antiterminator sequence (RAT) and a distant palindrome are both involved in sucrose induction of the *Bacillus subtilis* sacXY regulatory operon. *Microbiology* 141(Pt 11), 2921–2927. 10.1099/13500872-141-11-2921 8535520

[B49] Wagner E. G. Simons R. W. (1994). Antisense RNA control in bacteria, phages, and plasmids. *Annu. Rev. Microbiol.* 48 713–742. 10.1146/annurev.mi.48.100194.003433 7826024

